# Model resin composites incorporating ZnO-NP: activity against S. *mutans* and physicochemical properties characterization

**DOI:** 10.1590/1678-7757-2017-0270

**Published:** 2018-04-18

**Authors:** Natasha Lamego Brandão, Maristela Barbosa Portela, Luciane Cople Maia, Andréa Antônio, Vanessa Loureiro Moreira e Silva, Eduardo Moreira da Silva

**Affiliations:** 1 Universidade Federal Fluminense, Faculdade de Odontologia, Laboratório Analítico de Biomateriais Restauradores- LABiom-R, Niterói, Rio de Janeiro, Brasil; 2 Universidade Federal Fluminense, Faculdade de Odontologia, Niterói, Rio de Janeiro, Brasil; 3 Universidade Federal do Rio de Janeiro, Faculdade de Odontologia, Departamento de Odontopediatria e Ortodontia, Rio de Janeiro, Brasil

**Keywords:** Resin composite, Zinc oxide, Nanoparticles, S. mutans, Physicochemical properties

## Abstract

Although resin composites are widely used in the clinical practice, the development of recurrent caries at composite-tooth interface still remains as one of the principal shortcomings to be overcome in this field.

**Objectives:**

To evaluate the activity against *S. mutans* biofilm of model resin composites incorporating different concentrations of ZnO-nanoparticles (ZnO-NP) and characterize their physicochemical properties.

**Materials and Methods:**

Different concentrations of ZnO-NP (wt.%): E1=0, E2=0.5, E3=1, E4=2, E5=5 and E6=10 were incorporated into a model resin composite consisting of Bis-GMA-TEGDMA and barium borosilicate particles. The activity against *S. mutans* biofilm was evaluated by metabolic activity and lactic acid production. The following physicochemical properties were characterized: degree of conversion (DC%), flexural strength (FS), elastic modulus (EM), hardness (KHN), water sorption (W_sp_), water solubility (W_sl_) and translucency (TP).

**Results:**

E3, E4, E5 and E6 decreased the biofilm metabolic activity and E5 and E6 decreased the lactic acid production (p<0.05). E6 presented the lowest DC% (p<0.05). No significant difference in FS and EM was found for all resin composites (p>0.05). E5 and E6 presented the lowest values of KHN (p<0.05). E6 presented a higher Wsp than E1 (p<0.05) and the highest W_sl_ (p<0.05). The translucency significantly decreased as the ZnO- NP concentration increased (p<0.05).

**Conclusions:**

The incorporation of 2 – 5 wt.% of ZnO-NP could endow antibacterial activity to resin composites, without jeopardizing their physicochemical properties.

## Introduction

Since their development, the use of resin composites in the dental clinical practice has increased exponentially. However, irrespective of advantages such as the capacity to mimic the optical properties of the dental tissues and the on demand polymerization reaction, this class of restorative biomaterials still present limitations that impair its clinical performance. The accumulation of biofilm at the surface and the development of recurrent caries at the tooth-composite interface can be see as the principal shortcomings in this field^26^. Basically, resin composites consist of an organic matrix and inorganic filler particles bonded together by a bifunctional silane, photosensitizer substances, and additives such as stabilizers and pigments. In the last decade, however, dental scientists have been proposed the introduction of new components to improve different properties of these restorative biomaterials^25^. Nowadays, the development of bioactive composites capable of counterattacking the action of acidophilic bacteria, as well as the development of recurrent caries, seems to occupy the frontier of knowledge in this field^33^.

Methacrylate quaternary ammonium monomers (MQAM) chemically immobilized into the polymeric matrix have been used as a promising method to inhibit bacterial growth at resin composite surfaces. It is claimed that the great advantage of this approach is that the bioactive agent is not released from the matrix, which could contribute to the maintenance of the composites’ properties^8^. However, previous reports have shown that depending on the functionality and the concentration in which they are immobilized into the organic matrix, MQAMs can also reduce the mechanical properties^19^, and increase the water sorption of the composites^4^. Different bacteriostatic and bactericide chemicals, e.g., chlorhexidine, Ag salts and particles, oxides, and others, have also been tested to confer antibacterial activity to resin composites^7^ ^,^ ^25^. Unfortunately, these chemicals also jeopardized the composites’ physicochemical properties.

Recent studies have shown that resin composites incorporating ZnO presented antibacterial activity against *S. mutans and S. sobrinus* ^9^ ^,^ ^31^. However, these works have used commercially available resin composites to test this possibility. Since the exact composition of commercial resin composites is not known, it is plausible to claim that any chemical present in them could mask or interfere with the action mechanism of ZnO. Therefore, the goal of the present study was to evaluate the activity of a model resin composite incorporating different concentrations of ZnO-NP against *S. mutans* biofilm and to characterize their physicochemical properties. The hypotheses tested were: (1) the Model resin composites would present activity against *S. mutans* biofilm; and (2) the incorporation of ZnO-NP would not impair the physicochemical properties of the model resin composites.

## Materials and methods

### Formulation of model resin composites

**Figure 1:**
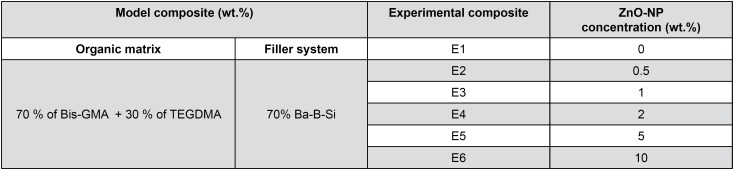
Composition of the experimental composites

A total of six experimental resin composites were formulated (Table 1). The organic matrix consisted of (wt.%): Bis-GMA and TEGDMA (70:30%) (Essthec Inc. Essington, PA, USA). The monomers were weighed in an analytical balance (AW 220, Shimadzu, Tokyo, Japan) and mixed with centrifuge at 1300 rpm for 1 min (SpeedMixer DAG 150FVZ-K, FlackTech Inc., Hauschild, Germany). Camphorquinone (0.5%) and ethyl N, N-dimethyl- 4-aminobenzoato - EDMAB (1.0%) were added and centrifuged at 1300 rpm for 1 min. Finally, 70% of barium borosilicate glass particles with an average size of 0.7 μm (Esstech, Inc., Essington, PA, USA) and ZnO-NP, 40-100 nm (AlfaAesar, Ward Hill, MA, USA), according to the tested concentrations (Figure 1), were incorporated, and the composites homogenized at 2400 rpm for 2 min.

**Table 1:** Mean ± SD of Degree of conversion (DC%), Flexural strength (FS), Elastic modulus (EM), Hardness (KHN), Water sorption (Wsp), Solubility (Wsl) and translucency (TP)

Composite	DC%	FS (MPa)	EM (GPa)	KHN (kgf/cm^2^)	W_sp_ (mg/mm^3^)	W_sl_ (mg/mm^3^)	TP
E1	68.6 ± 3.4^a^	83.3 ± 12.1	5.3 ± 1.2	67.2 ± 2.2^a^	24.8 ± 0.5^a^	2.8 ± 0.6^a^	21.6 ± 1.5^a^
E2	64.9 ± 1.3^ab^	77.9 ± 9.7	5.2 ± 0.9	64.4 ± 3.3^ab^	25.6 ± 0.9^ab^	2.4 ± 0.1^a^	12.7 ± 0.7^b^
E3	66.9 ± 1.0^a^	87.2 ± 6.9	5.3 ± 0.9	62.7 ± 2.1^a,b^	25.0 ± 0.6^ab^	3.0 ± 0.4^a^	9.9 ± 1.1^c^
E4	64.3 ± 1.5^ab^	86.4 ± 15.5	4.9 ± 1.1	59.8 ± 2.5^bc^	25.1 ± 0.4^ab^	2.8 ± 0.3^a^	6.0 ± 0.7^d^
E5	60.6 ± 2.0^b^	82.1 ± 8.8	4.6 ± 0.7	57.5 ± 2.9^cd^	25.6 ± 0.6^ab^	3.0 ± 0.2^a^	2.3 ± 0.5^e^
E6	51.0 ± 4.3^c^	90.9 ± 7.9	5.1 ± 0.6	54.4 ± 1.9^d^	26.1 ± 0.5^b^	3.9 ± 0.5^b^	0.7 ± 0.1^f^

In each column, means followed by different lowercase letter are statistically different (Tukey's HSD, p<0.05).

All the specimens in the present study were light-cured with a quartz-tungsten-halogen light unit (Optilux 501, Demetron Inc., Danburry, USA) using an irradiance of 650 mW/cm^2^ for 30 s (radiant exposure = 19.5 J/cm^2^).

### ZnO-NP size distribution analysis

ZnO-NP size distribution was performed using the NanoSight LM10 system (Malvern Instruments Ltd., Worcestershire, UK). The viscosity of the suspension medium was 0.95 cP, and the testing temperature was 22°C. Ten samples (n = 10) of the ZnO-NP were evaluated for 30 s, employing 30 frames per second, with camera shutter of 1 ms. The software used for capturing and analyzing the data was the NTA 2.3 Build 0025. The mean size determined by the NTA software corresponds to the arithmetic values of all the particles analyzed.

### Activity against *S. mutans* biofilm

Ten disk-shaped specimens (6.0 mm in diameter and 1.0 mm tick) were prepared for each experimental composite. Five of them were used in the biofilm metabolic activity assay and the other five used to inhibition of lactic acid production by the biofilm of *S. mutans.* The composites were inserted onto a stainless-steel mould, covered with a polyester strip and a glass slide, and light-cured from the top and bottom surfaces. Then, the specimens had their top and bottom surfaces wet polished with 1200 and 4000 grit SiC paper, sterilized with ethylene oxide and placed in a 24-well plate.

### *Streptococcus mutans* inoculation and biofilm formation

*S. mutans* ATCC 25175 (American Type Culture Collection, Rio de Janeiro, RJ, Brazil) was cultured in 20 ml of brain heart infusion (BHI) (Difco, Sparks, USA) broth supplemented with 2 % sucrose at 37°C in an anaerobic condition for 24 h. Afterwards, the bacterial suspension was adjusted to an optical density of 0.5 at 550 nm using an UV/Vis Spectrophotometer (Beckman Coulter DU^®^ 530, LifeScience, San Diego, CA, USA) in accordance to the McFarland scale (Biomérieux Brazil S.A., RJ, Brazil). The suspension was diluted by 1:100. Ten μl of this suspension was added into each well, containing a composite disk, with 2 ml of BHI broth supplemented with 2 % sucrose. The 24-well plates were incubated at 37°C in an anaerobic condition for 72 h. During the 3 days of the biofilm formation, the growth medium was changed every 24 h.

### MTT assay of metabolic activity

After 72, the composite disks were transferred to a new 24-well plate for MTT [3-(4,5-dimetylthiazol- 2-yl)-2,5-dyphenyltetrazolium bromide] evaluation, which is based on the enzymatic reduction of yellow tetrazolium into purple formazan. The specimens were rinsed three times with 1 ml of phosphate buffered saline (PBS, pH = 7.2) to remove cells not adhering to the biofilm, and 500 μl of MTT (1 mg/ ml) (Sigma - Aldrich, St. Louis, MO, USA) was added into each well and incubated at 37°C in a dark and in anaerobic condition for 1 h. Afterwards, 500 μl of dimethyl sulfoxide (DMSO) was added into each well and the plate was incubated at room temperature, in the dark for 20 min under gentle agitation. Then, 200 μl from each well was transferred to a 96-well plate and the optical density at 540 nm was measured in a microplate reader (ELx 808, BioTek Instruments, Winooski, VT, USA)^6^. A higher value of absorbance indicates more metabolic activity of *S. mutans* biofilm. The values of absorbance were subtracted from the blank (solution only, without biofilm) to be used in the statistical analysis.

### Lactic acid production

After 72 h of biofilm formation, the medium growth was removed and the composite disks were rinsed with 1 ml of PBS. Then, the specimens were transferred to a new 24-well plate and rinsed with 1 ml of buffered peptone water supplemented with 0.2 % sucrose (BPW). This medium was replaced with a fresh one and the plate was incubated at 37°C in an anaerobic condition for 3 h^6^. After the incubation period, the BPW solutions were used for lactic acid analysis, which was determined using a lactate dehydrogenase (LDH) reaction. Standard curves of the different concentrations of lactic acid were prepared in triplicate. The microplate reader (ELx 808, BioTek Instruments, Winooski, VT, USA) was used to measure the optical density at 340 nm.

### Scanning electron microscopy (SEM)

Two disks with *S. mutans* biofilm of each experimental composite were analysed by SEM. The disks were fixed in 2.5% glutaraldehyde supplemented with 3.7% sucrose for 1 h at room temperature, then gently washed with PBS to remove non-adherent biofilm. They were then fixed with 1% osmium tetroxide in 0.1 M of sodium cacodylate buffer containing 0.8% potassium ferrocyanide and 5mM of calcium chloride at pH 7.2 for 30 min at room temperature. Then, the disks were dehydrated in ascending ethanol series, submitted to critical point drying with CO_2_ and sputter-coated with Au-Pd. The specimens were mounted in aluminium stubs and viewed under SEM (JEOL-JSM-5310, Tokyo, Japan) operating in electron secondary mode. The images were taken at a magnification of 5,000x.

### Degree of conversion

Increments of each resin composite were inserted into a teflon mold (0.785 mm^3^) positioned onto an ATR crystal of the FT-IR spectrometer (Alpha-P/Platinum ATR Module, Bruker Optics GmbH, Ettlingen, Germany) and the spectra between 1600 and 1800 cm^−1^ were recorded with 120 scans at a resolution of 4 cm^−1^. Afterwards, the increments were light-cured and the spectra were recorded again (n = 3). The DC% was calculated from the equation (1):

$$DC\%=100\times \left[1-\frac{(aliphaticC=C)/(arometicC=C)cured}{(aliphaticC=C)/(aromaticC=C)uncured}\right]$$

Where R is the ratio between the integrated area of absorption bands of the aliphatic C=C bond (1638 cm^−1^) to that of aromatic C=C bond (1608 cm^−1^).

### Flexural strength and elastic modulus

Ten bar-shaped specimens were prepared for each experimental composite. The composite was bulk inserted into a stainless-steel mold with 10 mm × 2 mm × 1 mm dimensions, which was positioned upon a glass slide. After the mold was covered with a polyester strip and another glass slide, composites were light-cured, from the top and bottom sides using an overlapping regimen. The specimens were wet polished with 1200 and 4000 grit SiC paper, stored in distilled water at 37°C for 24 h and submitted to three- point bending test (DL 2000, EMIC, SP, Brazil) with 8.0 mm span, at a cross-head speed of 1 mm/min. The flexural strength (FS) in MPa and the elastic modulus (EM) in GPa were calculated using the equations (2) and (3), respectively:

$$FS=\frac{3/F}{2wh^{2}}$$

$$EM=\frac{Fl^{3}}{4wh^{3}d}$$

Where R is the ratio between the integrated area of absorption bands of the aliphatic C=C bond (1638 cm^−1^) to that of aromatic C=C bond (1608 cm^−1^).

### Microhardness (KHN)

Five disk-shaped specimens (2.0 mm in diameter × 3.0 mm in thickness) were prepared for each experimental composite. The specimens were embedded in epoxic resin, inside PVC cilinders, with the irradiated surfaces facing a glass plate. After the cure of epoxic resin, the irradiated surfaces were wet polished (DPU 10, Struers, Denmark) with 1200 and 4000 grit SiC paper (250 rpm/60 s in each paper) and five knoop indentations spaced of 500 μm were made in each specimen with 25 g load and dweel time of 15 s (Micromet 5104 / Full MHT software, Buëhler, Lake Bluff, IL, USA). The average of five indentations were take as the KHN for each specimen.

### Water sorption and water solubility

Five disk-shaped specimens (6.0 mm in diameter × 1.0 mm in thickness) were prepared for each experimental composite. After light-curing, the specimens were placed in a desiccator containing dehydrated silica gel and transferrred to an oven at 37°C. After 24 h, the specimens were daily weighed using an analytical balance with a precision of 0.01 mg (XP205, Mettler-Toledo Inc, Greifensee, Switzerland) until reach a constant mass *(m_1_)* (three consecutive days with a mass variation less than ± 0.01 mg). The thickness and the diameter of each specimen was measured at four points spaced equally at circumference using a digital caliper (MPI/E-101, Mitutoyo, Tokyo, Japan) and the volume (V) was calculated in mm^3^. The specimens were then individually immersed in 10 mL of distilled water at 37°C for seven days and weighed in the same analytical balance to obtain *m_2_.* Afterwards, the specimens were submmited to the same process described to obtain *m_1_* until a new constant mass was achieved *(m_3_).* Water sorption (W_sp_) and water solubility (W_sl_), in μg/mm^3^, were calculated using the equations (4) and (5), respectively:

$$W_{sp}=\frac{m_{2}-m_{3}}{V}$$

$$W_{sl}=\frac{m_{1}-m_{3}}{V}$$

### Translucency (TP)

Five disk-shaped specimens (8.0 mm in diameter × 2.0 mm in thickness) were constructed for each experimental composite. The color parameters were recorded according to the CIE L*a*b* system, against white and a black spectrophotometry ceramic standards (Konica Minolta Sensing Inc, Osaka, Japão), by using a spectrophotometer (model CM2600d, Konica Minolta Sensing Inc, Osaka, Japão). A D65 illuminant was used with 45° entrance angle and 0° observation angle geometry. The translucency parameter (TP) for each specimen was calculated using the following equation:

$$TP=\sqrt{\left(L_{W}^{*}-L_{B}^{*})+(a_{W}^{*}-a_{B}^{*})+(b_{W}^{*}-b_{B}^{*}\right)}$$

where the subscript B and W letters represent the measurements against the black and white backgrounds, respectively.

### Statistical analysis

The data were analysed using Statgraphics Centurion XVI software (STATPOINT Technologies, Inc., USA) by using one-way ANOVA and Tukey HSD's *post hoc* test. Regression analysis was performed to investigate the follow correlations: ZnO-NP (Wt.%) *vs.* DC%, and ZnO-NP (Wt.%) vs. TP. The analyses were performed at a significance level of α=0.05.

## Results

### ZnO-NP size distribution

**Figure 2:**
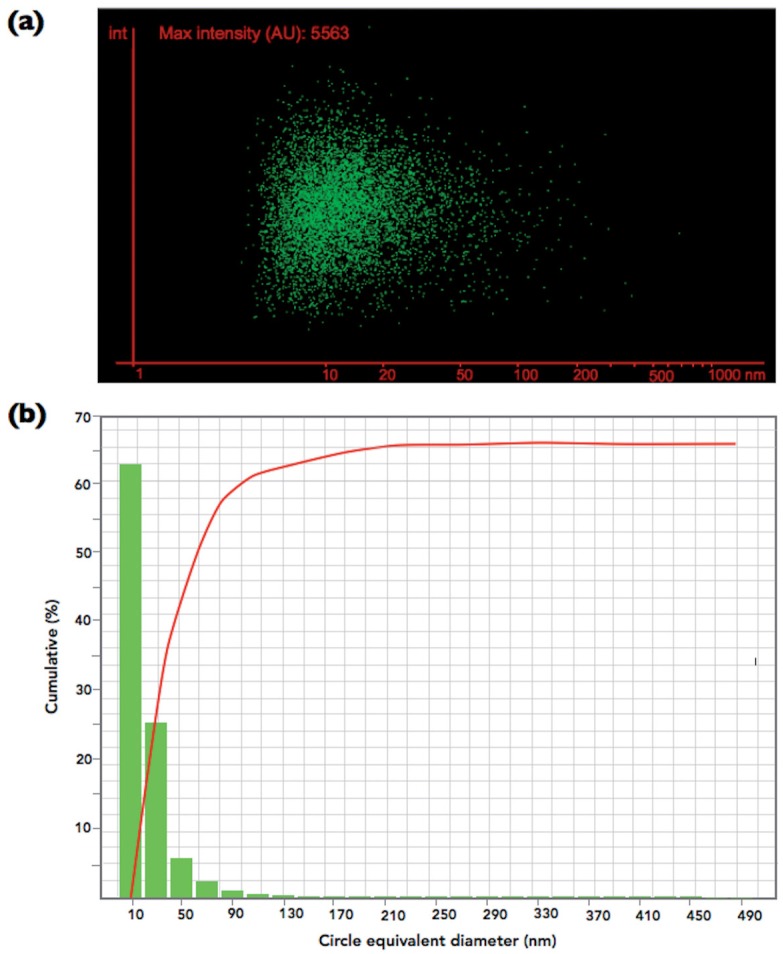
Size distribution of the ZnO-NP determined by NanoSight LM10 system. (a) image of the ZnO-NP dispersed in the test medium, and (b) histogram of the circle equivalent diameter (nm) of the ZnO-NP

The result of the size distribution analysis is presented in Figure 2. The ZnO-NP ranged from 7 to 810 nm, with a mean size of 12±19 nm.

### Activity against *S. mutans* biofilm

**Figure 3:**
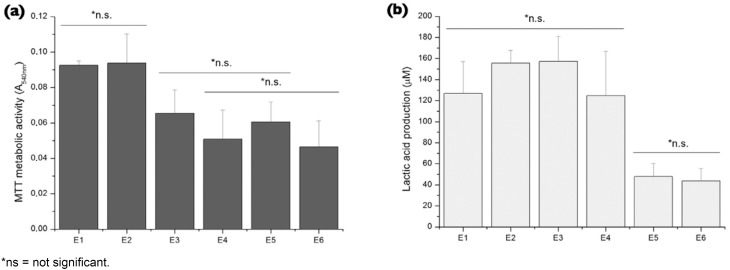
MTT assay of metabolic activity and Lactic acid production by *S. mutans* biofilm. Bars below each horizontal line are not statistically different from each other (*p* > 0.05)

The results of the MTT assay metabolic activity and the production of lactic acid by the *S. mutans* biofilm are depicted in Figure 3. The composites E3, E4, E5 and E6 decreased the metabolic activity of the *S. mutans* biofilm (*p*<0.05). Contrarily, the MTT absorbance of E2 was not statistically different from that of E1 (Figure 3a). Only the composites E5 and E6 reduced the production of lactic acid by the *S. mutans* biofilm (Figure 3b) (*p*<0.05).

**Figure 4:**
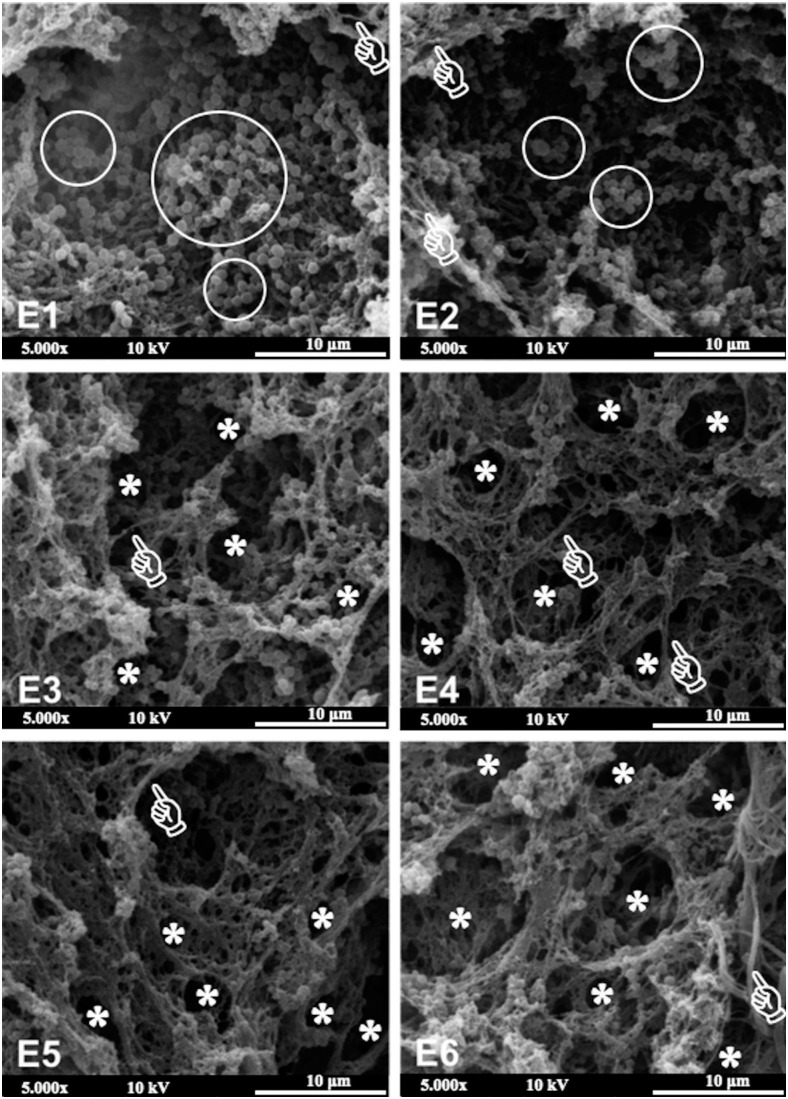
Scanning electron microscopy images (magnification 5,000x) of *S. mutans* biofilm on experimental composite surfaces

Representative SEM images of *S. mutans* biofilm on experimental composite surfaces are shown in Figure 4. Several spherical structures suggesting colonies of *S. mutans* (white circles) are evident on the surface of composites E1 and E2 (0 and 0.5 wt.% of ZnO-NP, respectively). A remarkable decrease in the number of these colonies were noted when the amount of ZnO-NP increased from 1 wt.% to 10 wt.% (composites E3, E4, E5 and E6). White pointers show the emergence of structures suggesting a connective extracellular polymeric substance (EPS) between bacterial colonies on all composite surfaces. Moreover, these EPS structures also showed vacuoles in areas with a low number of bacteria, which can be better viewed in composites E4, E5 and E6 (white asterisks).

### Physicochemical properties

The results of the physicochemical properties of all the composites are summarized in Table 1. The ZnO-NP concentration significantly influenced the DC%, with E6 presenting the lowest DC% (*p*<0.05). E1 E2, E3 and E4 presented similar DC% (*p* >0.05), while E5 developed an intermediate DC%, but was similar to those of E2 and E4 (*p* >0.05).

One-way ANOVA showed that the ZnO-NP concentration had no statistical influence on flexural strength (*p* = 0.1189) and elastic modulus (*p*=0.5786).

For hardness, one-way ANOVA showed that the ZnO-NP concentration had a statistically significant influence. The lowest hardness values were presented by E5 and E6 (*p*<0.05), although the value of E5 was similar to that of E4 (*p*>0.05). The hardness values of E1, E2 and E3 were not different from each other.

The water sorption was significantly influenced by the ZnO-NP concentration (*p*=0.0012), with E6 presenting a higher water sorption than E1 (*p*<0.05). The solubility of E6 was statistically higher than those of E1, E2, E3, E4 and E5 (*p*<0.05), which were not different from each other (*p*>0.05).

**Figure 5:**
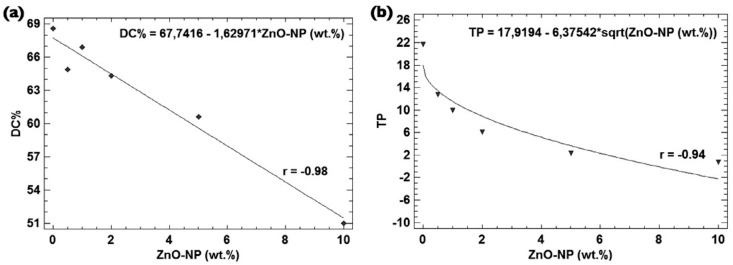
Linear Regression lines of (a) ZnO-NP concentration plotted against DC% (linear model), and (b) ZnO-NP concentration plotted against translucency (square-root X model)

Translucency (*TP*) was highly influenced by the ZnO-NP concentration: E1 > E2 > E3 > E4 > E5 > E6 (*p*<0.05). Regression analysis showed that the concentration of ZnO-NP strongly influenced the DC% (linear model; *r*=-0.98; *p*=0.0004 / Figure 5a), and translucency (square root-X model; *r*=-0.94; *p*=0.0057 / Figure5b).

## Discussion

Aydin Sevinç and Hanley^2^ (2010) were the first authors who tested the antibacterial activity of composites incorporating ZnO-NP, reporting a reduction of 80% in S. sobrinus biofilm growth when 10 wt.% of ZnO-NP was incorporated into a commercially available resin composite. More recently, Tavassoli Hojati, et al.^31^ (2013) also showed that *S. mutans* biofilm growth was significantly diminished with the increase in the ZnO-NP concentration. Although these two important studies have added interesting concepts regarding the effectiveness of ZnO-NP on biofilm growth inhibition, it is noteworthy that both incorporated the ZnO-NP particles into commercially available resin composites. Although the principal chemicals present in the composition of commercially available resin composites do not present antibacterial activity, it is reasonable to claim that some ingredients not disclosed by the manufacturers could interfere with the antibacterial mechanism of ZnO-NP. For example, in the study of Tavassoli Hojati, et al.^31^ (2013), ZnO- NP was added to Heliomolar Flow, a resin composite with ytterbium trifluoride in its composition. However, it was shown that Zn may enhance the glycolysis- inhibition effect of fluoride against *S. mutans* ^21^. Thus, in the present study the effectiveness of ZnO-NP was tested using a model resin composite with a known composition.

Thanks to its special abilities such as the production of EPS from the sucrose hydrolysis, which, in turns, facilitates the adherence of other bacteria onto tooth surfaces and contributes to the dynamic arrangement of the biofilm^32^, *S. mutans* is the first agent involved in bacterial colonization and biofilm growth. This was the rational behind using a single *S.mutans* biofilm in the present study. The MTT assay of metabolic activity was chosen to evaluate the activity of an experimental resin composite against *S. mutans* biofilm because it has been extensively used to evaluate the antibacterial potential of dental materials^6^ ^,^ ^7^.

Only the experimental composites with 1 to 10 wt.% of ZnO-NP presented activity against *S. mutans* biofilm (Figure 3a), results that corroborate those of Aydin Sevinç and Hanley^2^ (2010). Thus, the first null hypothesis of the present study was partially accepted. Although the manufacturer states that the ZnO-NP tested here fall in the range of 40 to 100 nm, the size distribution analysis showed that more than 60 % of these particles presented a circle diameter lower than 10 nm, with at about 90 % showing a maximum of 50 nm (Figure 2). Two pathways have ben described as the possible mechanisms for the antibacterial activity of ZnO. The first advocates that ZnO reacts with water from the environment, releasing Zn^2+^ into the growth media that may interfere with the bacterial metabolism by displacing Mg^2+^, which is extremely necessary to the enzymatic activity of the biofilm^23^. The second advocates that ZnO can also generate reactive peroxides that penetrate the membrane cell, causing damage and inhibiting bacterial growth^28^. Taking into account that both mechanisms involve release of active species from ZnO surfaces, it is clear that the high surface area to volume ratio of the 10 – 50 nm ZnO-NP particles used here (Figure 2) had influenced the behaviour of the experimental composites in the present study^34^. This thought is supported by the findings of Raghupathi, Koodali and Manna^27^ (2011). Analyzing the antibacterial activity of zinc oxide nanoparticles against S. *aureus (a gram positive bacteria as S. mutans),* these authors showed that this effect was size-dependent and better with the smallest particles (≈12 nm), which not only limited the growth of the bacterias but also killed them. Contrarily, the result of the composite with only 0.5 wt.% of ZnO-NP (E2) did not differ from that of E1. This means that this low concentration of ZnO-NP was unable to release sufficient Zn^2+^ or generated reactive peroxides in a quantity sufficient enough to act against *S. mutans* biofilm.

Fully hydrated biofilms are composed of cells (± 15 vol%) and EPS (± 85 vol%). Thus, after killing bacteria, more voids-like structures will be formed on the biofilm structure^10^. The analysis of Figure 4 shows that the number of *S. mutans* colonies (white circles) inversely decreased with the increase in the amount of ZnO-NP. In composites E3, E4, E5 and E6, it is clear the emergence of structures suggesting connective EPS between bacterial colonies (white pointers) and EPS vacuoles in areas with a low number of bacteria (asterisks). These aspects reinforce the antibacterial effectiveness of experimental composites with 1 to 10 wt.% of ZnO-NP. According to Weber, et al.^32^ (2014), these vacuoles represent spaces in the polysaccharide- rich EPS structure due to lower number of bacteria.

Caries starts with the progressive damage of the mineral structure of the dental hard tissues by organic acids produced by the cariogenic biofilm after fermentable carbohydrate intake^11^. Thus, quantifying the amount of lactic acid produced by *S. mutans* biofilm is also of great relevance in terms of antibacterial activity^6^ ^,^ ^35^. Only the composites with 5 and 10 wt.% of ZnO-NP significantly reduced the production of lactic acid by *S. mutans* biofilm (Figure 4), with the total amount of acid being three-fold less than that produced by the control composite. According to He, et al.^18^ (2002), this reduction in lactic acid production is due to the ability of Zn^2+^ to interfere with the bacterial glycolysis and be retained in plaque by electrostatic interaction, which may provides a prolonged bacteriostatic effect.

Although there is no sound evidence about the real influence of DC% on the clinical performance of resin composite restorations, it is known that properties such as hardness, strength, and solubility are directly related to this response^13^ ^,^ ^17^. Also, based on a strong negative correlation (r=0.91) between the degree of conversion (55 to 65%) and the abrasive wear of a hybrid resin composite *in vivo* ^14^, it is suggested that a DC% higher than 55% is desirable to high-stress bearing occlusal surfaces^30^. In the current study, only E6 presented a DC% below 55%, suggesting that this composite could be not suitable to build posterior restorations. Contrarily, the DC% of the other composites, ranging from 60.6 to 68.6% (Table 1), nicely agrees with others obtained from commercially available resin composites^1^ ^,^ ^36^, and falls in the hypothetical range capable of promote suitable clinical performance (55 – 65%). Irrespective of this, a strong negative correlation (-0.98) was found between ZnO-NP wt.% and DC% (Figure 5a). This result is in accordance with a previous study^3^, which showed that the higher the concentration of ZnO-NP, the lower the degree of conversion of experimental adhesives. Most probably, the decrease in the translucency of the composites with higher content of ZnO-NP (Figure 5b), due to the dissimilarity between the refractive index of the Bis-GMA:TEGDMA (70:30) blend (1.52)^29^, and that of ZnO-NP (2.02)^5^, could explain this finding.

As in the present study, Tavassoli Hojati, et al.^31^ (2013) also showed that the flexural strength was not affected by the incorporation of ZnO-NP. On the other hand, in that study the elastic modulus of composites with ZnO-NP were significantly higher than those of the control composite. As FS and EM are properties that involve a 3-dimensional material behavior, which is dependent on the material microstructure, it is assumed that in the current study the ZnO-NP reached a good dispersion inside the composites^15^ ^,^ ^31^, thereby not jeopardizing FS and EM (Table 1). This thought is supported by the findings of Barcellos, et al.^3^ (2016), who showed that the incorporation of ZnO-NP did not impair the flexural strength neither the flexural modulus of an experimental model dentin adhesive.

Microhardness suffered a significant decrease with the increase of ZnO-NP concentration (Table 1). This result is in agreement with that of Garcia, et al.^15^ (2016), who showed that the incorporation of ZnO nanoparticles significantly decreased the hardness of a experimental adhesive, and could be linked to the ZnO-NP hardness itself being lower than that of barium borosilicate glass particles^22^. Additionally, the decrease of DC% could also explain this result. This possibility corroborates the results of previous studies that showed a positive relationship between DC% and the microhardness of unfilled resins^12^ and resin composites^16^.

Although ISO4049 standard establishes specimens with 15 mm in diameter and 1 mm thick to evaluate W_sp_ and W_sl_, in the current study, we used specimens with 6 mm in diameter and 1 mm thick. This was done in order to allow the specimen to be light-cured in one step, which is more in conformity with clinical practice ^17^. The water sorption (24.4 to 26.1 μg/mm^3^) and the water solubility (2.4 to 3.9 μg/mm^3^) obtained in the current study (Table 1) are in agreement with the maximum values established by the ISO 4049 standard: W_sp_ ≤ 40 μg/mm^3^ and W_sl_ ≤ 7.5 μg/mm^3^ ^20^, suggesting that the composites formulated here may be resistant to hydrolytic degradation. E6 presented the highest solubility (3.9 μg/mm^3^), while the other composites did not differ from each other (Table 1). As ZnO is an amphoteric oxide insoluble in water and alcohols, this behaviour can be only explained through the lowest DC% developed by E6 (Table 1). This thought is supported by previous studies that showed good relationships between the degree of conversion and solubility of different resin-based materials^17^ ^,^ ^24^. Because the degree of conversion, hardness, water sorption, water solubility, and translucency were influenced by the different amounts of ZnO-NP, the second research hypothesis of the current study was rejected.

## Conclusion

Although the translucency was negatively affected by ZnO-NP, it is reasonable to conclude that the incorporation of 2 to 5 wt.% of ZnO-NP may endow antibacterial activity to resin composites, without jeopardizing their physicochemical properties. Irrespective of these findings, aspects such as the effect of pH-cycling, *in situ* simulations, and long-term evaluation must be addressed in future investigations.
